# Focus on Causality in ESC/iPSC-Based Modeling of Psychiatric Disorders

**DOI:** 10.3390/cells9020366

**Published:** 2020-02-05

**Authors:** Anke Hoffmann, Michael Ziller, Dietmar Spengler

**Affiliations:** Department of Translational Research in Psychiatry, Max-Planck Institute of Psychiatry, 80804 Munich, Germany; hoffmann@psych.mpg.de (A.H.); michael_ziller@psych.mpg.de (M.Z.)

**Keywords:** patient-specific iPSCs, schizophrenia, bipolar disorder, copy number variation, common variation, (epi-) genomic editing, massively parallel reporter assays

## Abstract

Genome-wide association studies (GWAS) have identified an increasing number of genetic variants that significantly associate with psychiatric disorders. Despite this wealth of information, our knowledge of which variants causally contribute to disease, how they interact, and even more so of the functions they regulate, is still poor. The availability of embryonic stem cells (ESCs) and the advent of patient-specific induced pluripotent stem cells (iPSCs) has opened new opportunities to investigate genetic risk variants in living disease-relevant cells. Here, we analyze how this progress has contributed to the analysis of causal relationships between genetic risk variants and neuronal phenotypes, especially in schizophrenia (SCZ) and bipolar disorder (BD). Studies on rare, highly penetrant risk variants have originally led the field, until more recently when the development of (epi-) genetic editing techniques spurred studies on cause-effect relationships between common low risk variants and their associated neuronal phenotypes. This reorientation not only offers new insights, but also raises issues on interpretability. Concluding, we consider potential caveats and upcoming developments in the field of ESC/iPSC-based modeling of causality in psychiatric disorders.

## 1. Introduction

Psychiatric disorders are among the leading cause of disability worldwide and place enormous demands on family and society [1,2]. In 2015, ~381 million (m) people were affected by major psychiatric disorders (~300 m by major depression (MD), ~60 m by bipolar disorder (BD), and ~21 m by schizophrenia (SCZ) as compared to ~35 m affected by cancer [3]). Demographic aging is expected to further heighten these numbers and the need for improvements on diagnosis, therapy, and prevention. Yet, in contrast to other common medical conditions like diabetes mellitus, no conclusive disease mechanism is established and remarkably little is known about cause-effect relationships in psychiatric disorders. This lagging reflects the intricate complexity of the brain, its inaccessibility in life, the lack of neuropathological clues to disease mechanism, and an inadequate toolkit until recently. Presently, diagnosis of psychiatric disorders relies on patients’ self-report and physicians’ observation of cognitive and behavioral symptoms rather than on direct measurement of an etiological factor. Conceptually, psychiatric disorders are complex syndromes in which quantitative deviations from health cut across historically-grown diagnostic boundaries [4].

In contrast to aforementioned uncertainties, there is, however, conclusive evidence on the heritability of psychiatric disorders. Estimates from family and twin studies suggest that heritability is high, especially for SCZ and BD, [5], and more broadly, implicate that risk for psychiatric disorders is encoded for in the human genome. Progress on high-throughput genotyping and genome-wide association studies (GWAS) enables the identification of genetic variants that increase the risk of SCZ, BD, and MD, among other psychiatric disorders (for recent review see [5,6]). These variants consist of common single nucleotide polymorphisms (SNPs) that conspire with rare gene-coding mutations and structural variants (e.g., copy number variations, (CNVs) and translocations) in the mediation of genetic risk. Although each of the disease-associated common SNPs confers on its own only tiny effects, they explain in aggregate a significant part of heritability. Disease-associated SNPs are also shared significantly between different psychiatric disorders, such as SCZ, BD, and MD, indicating that these three conditions are deeply interconnected at the genetic level [7]. 

**Figure 1:**
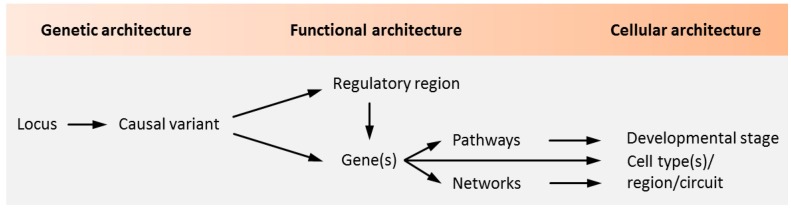
Causality in psychiatric disorders. Genetic studies identify loci containing sequence variants associated with psychiatric disorders. The aggregate of these variants makes up the genetic architecture of the disease. Genetic variants in coding regions are directly assignable to genes and can encode for altered protein function. A causal role of the coding variant is inferred once altered protein function is implicated in the pathogenesis of a psychiatric disorder. As most loci are non-coding, genetic variation in regulatory regions and the genes they regulate need to be experimentally defined. In light of the polygenetic architecture of psychiatric disorders, candidate regulatory regions and genes need to be investigated in the context of biological pathways and networks. These unfold during specific developmental stages in specific cell types to shape brain circuitries underpinning behavior, cognition, and mood among other brain functions that are impaired in psychiatric disorders. Adapted from [5], attributable license 4703001102503.

Notwithstanding the aforementioned progress on the polygenetic nature of psychiatric disorders, our insight into the causal role of genetic variation and risk genes is still in its infancy (Figure 1).

High-risk CNVs typically comprise large chromosomal regions hosting a multitude of genes potentially involved in the disorder. Likewise, most disease-associated lead SNPs (so-called index SNPs) fall outside gene coding regions and flag genomic regions that encode dozens, or even hundreds of additional linked SNPs. These SNPs cosegregate with the trait, mainly due to linkage disequilibrium (LD), and pose a major challenge to infer the potential causal role of individual SNPs (potentially causal SNPs and genes are referred to hereafter as credible SNPs and genes, respectively). Fine-mapping techniques, integration with epigenomic data, deep learning techniques, and GWAS on large populations improve nucleotide resolution; however, pinpointing the actual causal SNPs remains tedious. This limitation applies equally to the conundrum of which of the credible genes within the lead SNP-tagged genomic region actually contributes to the pathogenesis of psychiatric disorders.

To address this dilemma, integrative network and pathway level analyses were applied to predict different biological functions upon which disease-associated SNPs and genes potentially converge, including ion channel activity, synaptic plasticity, nervous system development, and neuronal differentiation [8,9]. A complementary line of evidence suggests that psychiatric risk loci are enriched in genes that are expressed in specific neuronal tissues and cell types from early development to adulthood [10,11,12,13,14,15,16]. Fetal gene expression from SCZ, but also BD risk loci, concurs with previous epidemiological and clinical evidence that early neurodevelopmental deviations could give rise to a vulnerable brain that converges with aberrant adolescent maturation processes on the manifestation of psychopathology [17]. Taken together, these studies associate polygenic risk for psychiatric diseases with pathways, cell types, and time windows that are most vulnerable to the combined action of these genetic variants (Figure 1), but do not define causal relationships between single genetic variants and cellular functions.

In this paper, we scrutinize how embryonic stem cells (ESCs) and the advent of patient-specific induced pluripotent stem cells (iPSCs) have opened new opportunities to investigate genetic risk variants in living disease-relevant cells. We focus on the question of how this progress has contributed to the analysis of causal relationships between genetic risk variants and neuronal phenotypes, especially in SCZ and BD. Studies on rare, highly penetrant risk variants have originally led the field, until more recently when the development of (epi-) genetic editing techniques spurred studies on cause-effect relationships between common SNPs and genes and their associated neuronal phenotypes. Concluding, we address current caveats and future needs for dissecting causal relationships between risk variants and cellular functions in the field of polygenic psychiatric disorders.

## 2. The Rationale of ESC/iPSC-Based Modeling of Psychiatric Disorders

ESCs, and even more patient-specific iPSCs, allow for overcoming a number of roadblocks that severely hampered studies on cause-effect relationships in psychiatric disorders: the unavailability of brain tissue from live patients and the limited value of postmortem tissue to inform on early disease processes that are of particular interest to timely prevention. Contrary to postmortem brain tissues, ESCs/iPSCs are neither inflicted by secondary alterations due to disease course, therapy, or patients’ life history and provide unlimited access to cell populations ranging from neural stem cells (NSCs) to neural progenitor cells (NPCs) to neurons [18].

In this manuscript we refer to NSCs as cells with the capacity to undergo self-renewing divisions that give rise to additional NSCs with the same properties, and divisions that produce daughter cells that differentiate into multiple cell types [18]. NPCs derive from NSCs and are the progenitor cells of the central nervous system (CNS) that produce many, if not all, of the glial and neuronal cell types that populate the CNS. NPCs do not generate the non-neural cells that are also present in the CNS, such as the cells of the immune system. NPCs are present in the CNS of developing embryos; but are also found in the neonatal and mature adult brain, and therefore are not strictly ESCs. After the onset of neurogenesis, NPC types in the mammalian neocortex are classified according to cell polarity, the presence of ventricular contact, and the location of mitosis [18]. In general, embryonic NPCs have more potential than NPCs in the adult brain. NPCs can be generated in vitro by differentiating ESCs or induced pluripotent stem cells (iPSCs). Importantly, gene expression profiling from embryonic to aged postmortem brains supports that differentiating ESCs/iPSCs tightly track progression from early embryogenic to perinatal stages in vivo and develop into neuronal and glial cells of varying maturity [19,20,21,22,23,24]. 

Genetic engineering allows for the introduction of single, or at best a handful of, genetic variations associated with disease risk in human ESCs. Yet, such modifications cannot recapitulate the polygenic risk architecture of psychiatric disorders present in patient-specific iPSCs (not to mention serious ethical constrains on the productions of human ESCs). Likewise, transgenic animal models, while informative for studying the role of single or a few human risk genes, are limited in recapitulating common variants from non-coding, weakly conserved, gene regulatory regions, and even less so on a genome-wide scale. By contrast, iPSCs capture the donor’s original genotype and enable investigation on a wide range of psychiatric disorders in vitro through differentiation toward disease-relevant neuronal and glial cells, in which the patient-specific genetic architecture is preserved. Nowadays, iPSCs are derived routinely from virtually any cell type, most commonly from blood or skin biopsies by delivery of a cocktail of reprogramming-factors [25]. Since iPSCs portray genetically encoded molecular and cellular phenotypes from early development, they may better inform on genetic predisposition to psychiatric disorders than the disease state itself [26]. Hence, caution should be applied to extrapolate from iPSC-derived cell stages to those in patients, a reservation that also applies to ESC-based modeling.

## 3. ESC/iPSC-Based Modeling of Structural Variants

CNVs are important risk factors in the onset and treatment resistance of SCZ and BD [27,28,29]. These genetic variants typically arise from region-specific, repetitive DNA sequences dubbed low copy repeats (LCRs). Recent meta-analysis (21,094 patients with SCZ versus 20,227 healthy controls) found in a small fraction (~1.4%) of the patients a genome-wide significant association with CNVs [30]. These CNVs comprised eight loci: 1q21.2, 2p16.3 (see below), 3q29, 7q11.2, 15q13.3 (see below), distal 16p11.2, proximal 16p11.2, and 22q11.1. Though risk CNVs explain only little of the variance in SCZ liability when compared to common risk loci (0.85% versus 3.4%), they strongly impact SCZ risk relative to common variants (e.g., CNV at 2p16.3 (encoding for *NRXN1*): odds ratio (OR) ~14.4 versus common variants: OR <1.3) [29]. Very rare de novo CNVs, also known as private CNVs, are additionally identified in single individuals or families. Though such private variants do not inform on general risk in psychiatric disorders, they can offer valuable molecular leads to pathways underpinning pathogenesis. Both categories of de novo CNVs are discussed in this section with major findings summarized in a tabular format (Table 1).

### 3.1. CNV Risk Variants at 2p16.3 Associate with Synaptic Transmission

Most heterozygous CNVs at 2p16.3 affect only *NRXN1* (*Neurexin-1*) due to its large size and are the most frequent single-gene mutation (~0.18%) in SCZ [46]. Beyond SCZ, 2p16.3 microdeletions are also associated with developmental delay (speech delay, behavioral abnormalities, mild dysmorphic features) or exist in healthy parents and siblings. These varying clinical outcomes reflect the pleiotropic nature of CNVs and dependency on genomic context [29]. Additionally, common variations in *NRXN1* are implicated in the responsiveness to antipsychotic or antidepressant treatments [47].

The neurexin gene family (*NRXN1*-*3*) encodes for proteins with an essential role in synapse organization and synaptic transmission [48]. Through their interaction with various pre- and postsynaptic proteins they facilitate the exocytosis of neurotransmitters and form the inter-synaptic complex that coordinates the generation, specification, and maturation of synapses. Compatible with these findings, loss of function studies in mice suggest that *Nrxn1* plays a role in memory formation, and social and behavioral tasks [47].

*NRXN1* encodes for 23 exons straddling 1.12 Mb and is one of the largest genes in the human genome. Two promoters localize separately at the 5′ end of *NRXN1* or downstream from exon 17 with resultant RNAs translated into either NRXN1α or NRXN1β isoforms. Genetic variations, especially microdeletions in SCZ, occur more frequently in the upstream than the downstream promoter.

**Figure 2:**
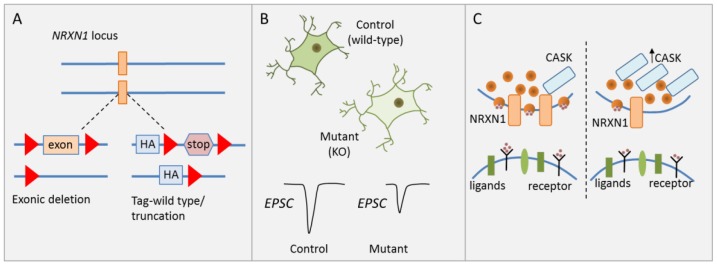
*NRXN1* regulates synaptic transmission. (**A**) Schematic drawing of heterozygous *NRXN1* mutations consisting of either a conditional exonic deletion or a conditional truncation. (**B**) Neural development and morphological features were unaffected in human ESC-derived glutamatergic iNS carrying wild-type alleles or either heterozygous *NRXN1* mutations. Presynaptic neurotransmitter release was reduced in iNs with *NRXN1* deletions and associated with reduced stimulated EPSC amplitude. (**C**) CASK (calcium/calmodulin-dependent serine protein kinase) protein levels were significantly increased in neurons carrying either *NRXN1* mutation. Neurexins interact with CASK, a cytoplasmatic scaffolding protein, in the regulation of presynaptic neurotransmitter release. Increased CASK protein concurred with reduced presynaptic neurotransmitter release. Adapted from [31], attributable license 4680770115438.

To investigate the causal role of heterozygous *NRXN1* deletions in human neuronal cells of a defined genetic background, Pak et al. [31] generated isogenic human ESC lines carrying two kinds of heterozygous *NRXN1* mutations: (i) a conditional exon deletion disrupting both neurexin-1α and -1β via a frameshift, and (ii) a conditional truncation of neurexin-1α and -1β via introduction of a stop codon leading to fast degradation of the truncated protein (Figure 2A). 

Since cellular heterogeneity during neuronal differentiation confounds the analysis of cell-type specific effects [49], forced expression of the transcription factor (TF) Neurogenin-2 was used to induce a homogenous, well-characterized population of excitatory cortical neurons (hereafter referred to as induced neurons, iNs). Synapse numbers, dendritic arborization, and electrical properties were unaffected by either heterozygous *NRXN1* mutations in excitatory iNs (Figure 2B). By contrast, a significant and selective decline in presynaptic neurotransmitter release was detected in excitatory iNs carrying the deletion. This decline is associated with a reduction in spontaneous miniature excitatory postsynaptic current (mEPSC) frequency and a reduction in stimulated EPSC amplitude (Figure 2B). Notably though, the latter parameter quickly recovered following a high-frequency stimulus train suggesting that this impairment applied only to the first stimulus, but not to release probability in general. In agreement with this hypothesis, heterozygous *NRXN1* mutations did not impact the size of the readily releasable neurotransmitter pool. Beyond cortical iNs, Pak et al. [31] collaborated the NRXN1 phenotype in a second less homogenous culture of neurons from ESC-derived NPC intermediates. 

A likely mechanism mediating impaired synaptic transmission is reduced Ca^2+^ influx, which can be compensated by accumulation of residual Ca^2+^ in a high-frequency stimulus train. To explore the molecular basis of this hypothesis, Pak et al. conducted a limited screen for expression changes in genes contributing to synaptic function, adhesion, and signaling. While mRNA levels of CASK (calcium/calmodulin-dependent serine protein kinase) were unaffected, CASK protein levels were significantly increased in neurons carrying either *NRXN1* deletion (Figure 2C). CASK is a cytoplasmatic scaffolding protein that interacts with neurexins, increasing the possibility that neurexin-1 controls CASK levels and possibly regulates neurotransmitter release as well [50]. 

Taken together, Pak et al. [31] established the causal role of a high-risk CNV in SCZ: disruption of *NRXN1* led to impaired presynaptic neurotransmitter release in vitro and was associated with a decline in mEPSC frequency and stimulated EPSC amplitude. This study is the first of its kind to introduce a technological platform for analyzing the causal and biological significance of gene mutations in psychiatric disorders in disease-relevant cell types in vitro, with the underlying conception that the effects from these mutations are likely to be pathogenic in vivo. 

As noted, heterozygous exonic deletions in *NRXN1* present with variable penetrance and diverse clinical symptoms for reasons poorly understood. One possible explanation for this variability is extensive alternative splicing of the human and mouse genes with more than 200 isoforms detected in the mouse prefrontal cortex by single-cell sequencing (sc-RNAseq). This process occurs in a partly cell type-dependent manner, raising the prospect that the resultant protein isoforms may distinguish between post-synaptic binding partners to regulate specific synaptic function [47]. To explore whether this process contributes to neuropathology in carriers of heterozygous intragenic *NRXN1* deletions, Flaherty et al. [45] established iPSCs from four carriers with major psychiatric disorders, three with early onset, and three healthy controls. Advanced sequencing methods were applied to integrate targeted long- and short-read sequencing data, and to establish *NRXN1a* isoform catalogues in human postmortem brain and across iPSC-derived isogenic glutamatergic or GABAergic (γ-aminobutyric acid) iNs. Interestingly, neurons derived from patient-specific iPSCs showed significant perturbations in the expression of *NRXN1a* isoforms, including decreased levels of many wild-type isoforms, and de novo expression of patient-specific mutant *NRXN1a* isoforms. This perturbation was associated with impaired neuronal activity and immature gene expression profiles. Concurrently, synapse formation was delayed and did not evolve along the same developmental trajectory as control-derived iPSC neurons. Notably, this deficit was rescued by overexpression of individual wild-type NRXN1 isoforms, but only in case mutant isoforms were not expressed. Contrariwise, overexpression of individual mutant isoforms in wild-type neurons significantly decreased neuronal activity, indicating a possible dominant-negative function of heterozygous *NRXN1* deletions.

In sum, these findings suggest that alterations in the repertoire of *NRXN1α* isoforms in carriers of heterozygous *NRXN1* deletions underpin perturbed synaptic function and neuronal maturation during brain development and thus may increase the risk for later major psychiatric disorders. While this hypothesis offers a plausible explanation for the variable penetrance and clinical presentation of heterozygous *NRXN1* deletions owing to quantitative effects on isoform expression, including potentially dominant negative variants, it does not answer the ‘chicken or egg’ question of which came first: Does aberrant neuronal maturation inflict synapse function, or does aberrant synapse function through activity-dependent mechanism inflict neuronal maturation?

### 3.2. CNV Risk Variants at 15q11.2 Associate with NSC Adherens Junctions and Cell Polarity 

Different CNVs within the proximal arm of chromosome 15 (15q11.2-q13) associate with an increased risk for SCZ, ASD (autism spectrum disorder), and neurodevelopment-related disorders [51]. Among three proximal breakpoints termed BP1, BP2, and BP3, the BP1-BP2 microdeletion at 15q11.2, with a prevalence of 0.57%–1.27%,is one of the three most frequent CNVs for SCZ and increases risk two- to four-fold [52]. In neuroimaging studies, this microdeletion affects brain structure in a pattern matching both that observed during first-episode psychosis in SCZ and that of structural correlates in dyslexia [53]. Four protein-encoding genes localize to this region: *TUBGCP5*, *CYFIP1*, *NIPA1*, and *NIPA2* [51]. *TUBGCP5* (tubulin gamma complex associated protein 5) is necessary for microtubule nucleation and implicated in ADHD (attention deficit hyperactivity disorder) [54]. Mutations in *NIPA1* (non-imprinted in Prader-Willi/Angelman syndrome 1 gene) cause autosomal dominant hereditary spastic paraplegia. *NIPA1* and *NIPA2* (non-imprinted in Prader–Willi/Angelman syndrome 2 gene) regulate Mg^2+^ transport in neurons and the kidney, respectively. CYFIP1 (cytoplasmatic FMR1-interacting protein) is a binding partner of FMRP (fragile X mental retardation protein) and interacts with Rac1, a RHO (Ras homologue) GTPase involved in cytoskeleton modulation, neuronal polarization, axonal growth, and differentiation [51]. Biochemical studies further suggest that CYFIP1 regulates the WAVE complex controlling Arp2/3-mediated actin polymerization and membrane protrusion in non-neuronal cells.

**Figure 3:**
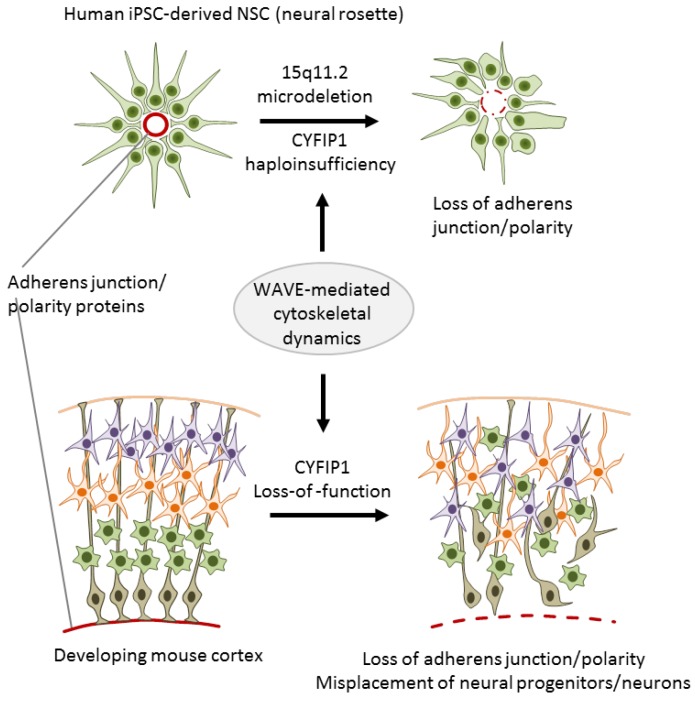
The 15q11.2 microdeletion associates with impaired adherens junctions and cell polarity. Neural rosettes derived from iPSCs of donors with the microdeletion and childhood onset of schizophrenia show reduced adherens junctions formation and apical–basal polarity when compared to age-matched healthy donors without the microdeletion (top). During corticogenesis, Cyfip1 knockdown disrupted mitosis and the destination of radial glial progenitor cells (bottom). Adapted from [32], attributable license 4685390685838.

To investigate the role of 15q11.2 haploinsufficiency in SCZ, Yoon et al. [32] generated iPSC lines from three carriers with childhood onset of SCZ (COS) and five healthy individuals without the microdeletion. COS is clinically continuous to adult onset SCZ though its course is more severe and manifests more frequently neurodevelopmental deviations and delay. Upon differentiation, iPSC-derived neural rosettes from cases showed reduced adherens junctions formation and apical–basal polarity relative to controls (Figure 3). 

Both features are regulated by the actin cytoskeleton that is under the control of the WAVE complex. In agreement with non-neuronal cells, CYFIP1 also regulated the WAVE complex in NPCs as evidenced by immunoprecipitation experiments [32]. As a result of this regulatory cascade, NPCs from carriers with COS exhibited a specific defect in WAVE complex stabilization. Importantly, lentiviral complementation of CYFIP1 rescued the defect in adherens junctions and apical polarity in NPCs from carriers with COS, whereas CYFIP1 knockdown reduced WAVE2 protein levels in NPCs from healthy controls. 

In an independent approach, Yoon et al. [32] further showed that cyfip1 is necessary to sustain adherens junctions and apical polarity of NSCs in a developing mouse cortex: Cyfip1 knockdown disrupted mitosis and the destination of radial glial progenitor cells (RGCs) in the developing cortex (Figure 3). This deficit subsisted in intermediate progenitor cells (IPCs), the direct progeny of RGCs, as well as in glutamatergic projection neurons, and exhibited altered stratification of projection neurons and cortical layer formation. 

Overall, Yoon et al. [32] firstly introduced integrated iPSC-based modeling by using human iPSCs as a leading in vitro discovery tool whose results were validated in transgenic mice. This integrated approach supports that CYFIP1 controls the formation of adherens junctions and apical polarity in vitro and impacts RGC function, corticogenesis, and cortical layer formation in vivo. Though this study did not explicitly rule out a role of the remaining genes localized at the 15q2 microdeletion, the combination of complementary in vitro and in vivo experiments allows assigning *CYFIP1* haploinsufficiency a causal role in SCZ. Given that defects in cortical patterning are thought to contribute to SCZ and neurodevelopment-related disorders [55], a salient explanation for the pathogenic role of CNVs at 15q11.2 in SCZ is that *CYFIP1* haploinsufficiency causally affects corticogenesis.

### 3.3. Private CNVs at PCDH15 and RELN Impact Dendrite and Synapse Formation

Increased rates of de novo CNVs were originally described in SCZ, ASD, and developmental delay, but also more recently in BD [27]. De novo CNVs occurred in 1.5% of controls, 2.2% in BD, and in 4.3% in SCZ and were more likely in sporadic or early onset cases. One private CNV mapped to 10q21.2 in one patient with BD and caused a heterozygous deletion of *PCDH15* (protocadherin 15). In support of this finding, a similar-sized exonic deletion of *PCDH15* was detected in an another patient with BD [56]. Furthermore, recent GWAS meta-analysis suggests a significant association between *PCDH15* SNP variants and personality traits in psychiatric disorders [57]. 

Clustered protocadherins (Pcdhs) play key roles in the generation of cell surface structural diversity. The ability of individual neurons to distinguish between themselves and neurites from other neurons (self-avoidance) is regulated by highly specific homophilic interactions between Pcdh complexes that direct neurite repulsion rather than adhesion and maximize target coverage (tiling) [58]. These processes play a central role in the development of complex neural circuits. Consistent with this view, variation in *Pcdhs* weakening homophilic interactions is associated with wiring defects and abnormal behavior in mice. 

Similar to BD, two patients with SCZ were recently identified with a private CNV causing heterogeneous exonic deletions in *RELN* (Reelin) [56,59]. This extracellular matrix protein is secreted by Cajal–Retzius cells and a subpopulation of GABAergic interneurons. *RELN* plays a critical role for proper migration and positioning of cortical neurons during embryonic and early postnatal stages. As noted above, aberrant neuronal migration and laminar formation during corticogenesis is implicated in psychiatric and neurodevelopment-related disorders [55]. Variations in *RELN* were also found to associate with SCZ, BD, and ASD [60] and *Reln* haploinsufficiency in mice caused cognitive impairment and a decline in GABAergic neurons [61]. 

To investigate a causal role of *PCDH15* and *RELN* haploinsufficiency in the pathogenesis of psychiatric disorders, Ishii et al. [33] generated iPSCs from each two patients with BD and SCZ carrying heterozygous *PCDH15* and *RELN* deletions, and from five healthy controls. Although embryoid body (EB) capacity of differentiation into the three germ layers was unaffected, neurite extension of the EB clusters was stunted in cases indicating an early differentiation deficit. In glutamatergic or GABAergic iNs, *PCDH15* and *RELN* expression was undistinguishably upregulated in case/control groups indicating the existence of compensatory mechanisms in patient cells, even though, gene expression profiling identified a number of differentially expressed genes (DEGs) in neurons from cases relative to controls, which were especially enriched for genes with a role in cell adhesion and neural function. Consistent with this result, mature dendrites were significantly shorter in both glutamatergic and GABAergic iNs from cases relative to controls. Likewise, synapse numbers were less for cases relative to controls for both cell types. However, spontaneous neuronal activity was undistinguishable between case/control groups. A possible explanation of the latter result was that neurons from patients with BP or SCZ displayed higher sensitivities in AMPA (α-amino-3-hydroxy-5-methyl-4-isoxazolepropionic acid) or GABA receptor stimulation that might have sustained spontaneous activities in the presence of structural deficits. 

Having defined the phenotype of iPSC-derived neurons from patients with BD/SCZ carrying heterozygous *PCDH15/RELN* deletions, Ishii et al. [33] applied targeted genome editing (CRISPR/CAS9) (clustered regulatory interspaced short palindromic repeats/CRISPR-associated protein 9) to introduce patient-like mutations in two healthy control iPSCs. EBs derived from isogenic homozygous gene-edited iPSCs showed for either mutation unaffected germ layer formation ruling out a general differentiation defect. By contrast, mature dendrites of glutamatergic iNs from *PCDH15*-edited iPSCs, but not from *RELN1*-edited iPSCs, were significantly shorter than those from isogenic controls, while synapse numbers were unaffected under either condition. Moreover, in GABAergic iNs dendrite formation was only reduced in *PCDH15*-edited cells, while reductions in synapse numbers occurred only in *RELN1*-edited iPSCs. 

Taken together, iPSC-derived neurons from psychiatric patients with deletions in *PCDH15* or *RELN1* as well as from gene-edited isogenic controls share morphogenetic abnormalities, partly in a cell type-dependent manner. This finding suggests that very rare CNVs in *PCDH15* or *RELN1* contribute causally to brain pathology by affecting dendrite and synapse formation. A limitation of the present study [33] is the use of homozygous deletions in gene-edited control iPSCs raising the issue of gene dosage. Therefore, it would be interesting to know to what degree rescue of *PCDH15* or *RELN1* in patients’ iPSCs would normalize structural abnormalities. Independent support of a critical role of protocadherins in psychiatric disorders was recently obtained by the transplantation of iPSC-derived cortical inhibitory interneurons (cINs) from healthy controls and patients with SCZ into cerebral cortices of mice [62]. Transplants from either group developed undistinguishably into authentic functioning interneurons in vivo and gene expression profiling of in vitro differentiated cINs uncovered only few DEGs. Among these were a subgroup of protocadherin alpha genes (*PCDHAs)*, whose downregulation was associated with reduction in neurite branch numbers and neurite length in vitro. Independent transgenic mice and postmortem brain analysis further supported an early and cell-autonomous defect in cINs. Although credible SNPs in *PCDHAs* could not be identified owing a lack of statistical power, this study highlights the potential of phenotype-driven iPSC-based disease modeling for dissecting causal mechanisms in SCZ (see also [63]).

### 3.4. DISC1′s Role in Presynaptic Function and Neural Development

Nearly 20 years ago, a balanced (1;11)(q42.1;q14.3) translocation was hypothesized to segregate with major mental disorders, including SCZ, BD, and recurrent MD in a large Scottish family (Millar et al. 2000). Though some carriers were unaffected, all individuals with the aforementioned diagnoses carried the translocation that disrupted a gene dubbed DISC1 (disrupted in schizophrenia). Since then, no other family with this translocation has been identified and the role of *DISC1* in major psychiatric disorders is disputed [64]: Genome-wide linkage, CNV, and common variant analysis do not support *DISC1′s* role as a common bona fide risk gene in psychiatric disorders, but suggest that variations in *DISC1* affect only a few individuals, families, or ethnic groups [65]. 

In support of this view, Sachs et al. [66] identified an American family in which four members with major psychiatric disorders (two each with SCZ or MD) carried a 4-bp frameshift deletion of *DISC1* in the extreme 3′ end of exon 12. To explore the role of this deleterious *DISC1* mutation, Wen et al. [34] established iPSCs from the four affected and from two unaffected family members and differentiated them into glutamatergic forebrain neurons. Electrophysiological measurements showed presynaptic dysfunction and impaired synaptic transmission in mature neurons from cases relative to controls. To assess the causal role of the 4-bp deletion for this phenotype, Wen et al. corrected via gene editing the 4-bp deletion in one iPSC line carrying the mutated *DISC1* allele, and introduced additionally the 4-bp deletion into two control iPSC lines carrying the wild-type allele: one control iPSC line within the pedigree, and one unrelated to the pedigree to account for genetic background. Importantly, correction of the 4-bp deletion rescued the electrophysiological and synaptic defects in neurons derived from the isogenic patient iPSC line, whereas introduction of the 4-bp mutation recapitulated the phenotype from cases in both isogenic control iPSC lines. These results strongly support a causal role for the heterozygous *DISC1* deletion in the synaptic defect of iPSC-derived human neurons in vitro. 

Expression profiling revealed that DEGs between cases and controls were enriched in those implicated in ‘synaptic transmission’, ‘nervous system development’, ‘dendritic spine’, and ‘psychiatric/mental disorders’. Notably, these differences were recapitulated between forebrain neurons derived from isogenic iPSCs strengthening the causal link between DEGs and *DISC1* status. In a recent follow up study, Wang et al. [35] further showed that the physical interaction between mutated DISC1 and the TF ATF4 mediated the effects on aberrant gene expression and synapse function. In support of this hypothesis, ATF4 overexpression rescued aberrant phenotypes in iPSC-derived forebrain neurons carrying the 4-bp deletion.

Taken together, heterozygous mutations in *DISC1* cause impaired synapse function of iPSC-derived forebrain neurons in vitro. The elegant use of complementary isogenic iPSC lines strongly supports this conclusion and suggests that deleterious *DISC1* mutations are a defining cause in synaptic pathology in patients with major psychiatric disorders.

Beyond synaptic dysfunction, *DISC1* is also implicated in neural development. Srikanth et al. [36] established isogenic human iPSC lines from healthy donors carrying engineered disruptions of *DISC1* in exon 2, common to all isoforms, or near the site of the balanced translocation in exon 8. Targeting introduced preterminal stop codons, homozygous in the case of exon 2, and heterozygous in the case of exon 8, which led to a complete or halfway loss of a full-length DISC1 protein. Rosette-selected dorsal forebrain NPCs showed a reduced expression of cell-fate markers upon either *DISC1* interruption that persisted in mature neurons. In exon 2, but not in exon 8, interruption was also associated with premature differentiation and perturbed cortical layer marker expression. Consistent with these findings, genome-wide expression profiling of NPCs and neurons revealed a subtle dorsal shift in cell fate as result of *DISC1* interruption. 

In sum, disease-relevant *DISC1* mutations in gene-edited isogenic iPSC lines from healthy donors drove subtle shifts in dorsal fate with NPCs differentiating prematurely at the cost of ordered cortical layer formation in vitro.

More recently, Srikanth et al. [37] reexamined both kinds of *DISC1* mutations (i.e., the 4-bp frameshift in exon 12 and the heterozygous exon 8 deletion) in glutamatergic iNs. Mature neurons were derived from the respective isogenic wild-type and mutant iPSC lines. In this differentiation paradigm, both *DISC1* mutations caused subtle alterations in a subset of presynaptic proteins that converged on a significantly decreased expression of just four genes including *UNC5D*.

This gene, like other *UNC5* family members, encodes for a cell surface co-receptor for netrin-1 in conjunction with the netrin receptor DCC (deleted in colorectal carcinoma). Netrin-1, an extracellular secreted protein, controls axon guidance, synapse formation, and cell migration through repulsive cues during development. Consistent with these features, neurite outgrowth assays showed an early and persistent decrease in neurite length in neurons carrying either exon 8 or exon 12 *DISC1* mutations. Importantly, knockdown of *UNC5D* in wild-type neurons recapitulated the phenotype from *DISC1* mutant cells. Conversely, *UNC5D* activation via CRISPR/CAS9 in *DISC1* mutant neurons rescued neurite outgrowth.

In brief, different disease-relevant mutations in *DISC1* converge on subtle changes in presynaptic gene expression in cortical neurons. These changes underpin impaired neurite outgrowth in an *UNC5D*-dependent manner in vitro. All in all, findings from this [37] and previous reports [34] strengthen the hypothesis that *DISC1* mutations causally impact presynaptic function in vitro. Even though, we would like to emphasize again that their relevance to common psychiatric disorders remains controversial since no additional families carrying the mutations from the original index cases have been identified so far. 

### 3.5. SHANK3 Mutations Impact Neuronal and Synaptic Functions

The 22q13.3 deletion syndrome, also known as Phelan–McDermid syndrome (PMDS), is a rare neurodevelopmental disorder characterized by global developmental delay, intellectual disability, absent or delayed speech, and increased risk for ASD [67]. Most deletions emerge de novo, are paternally derived, and comprise non-recurrent breakpoints covering up to 9 Mb. Among the 90 genes locating to the terminal deletions, *SHANK3* is thought to present the most likely candidate gene for the neurobehavioral correlates of PMDS [67]. In support of this hypothesis, large-scale targeted resequencing and whole-exome sequencing identified *SHANK3* point mutations and indels in subjects diagnosed ADHD, intellectual disability, and SCZ [67,68]. 

The *SHANK* (SH3 and multiple ankyrin repeat domains) family comprises *SHANK1*, *SHANK2*, and *SHANK3* encoding for a family of proteins, which are highly enriched in a specific compartment of the postsynaptic membrane of excitatory synapses, known as postsynaptic density (PSD). This structure contains a network of scaffolding proteins among which SHANK3 functions as a master scaffolder by forming large sheets that may provide the platform for the assembly of the entire PSD complex [69]. By this virtue, SHANK3 is thought to play a critical role in synaptic transmission and plasticity.

To gain insight into the neuropathology of *SHANK3* disruptions, Shcheglotivo et al. [38] generated iPSCs from PMDS patients with ASD and differentiated them into functional forebrain neurons. Compared to neurons derived from control iPSCs, cases showed reduced SHANK3 expression, increased input resistance, and impaired excitatory transmission. The latter resulted from both a decline in excitatory synapses and glutamate receptor expression. By contrast, inhibitory synaptic transmission was unaffected in cases relative to controls. In support of SHANK3′s critical role in synaptic dysfunction, lentiviral complementation of SHANK3 restored excitatory synaptic transmission, but not input resistance, in case neurons. While this finding is consistent with SHANK3′s scaffolding function in the PSD, it does not explain the increased input resistance in iPSC-derived neurons from patients with PMDS, nor does it implicate a causal role of SHANK3 in this process.

To clarify this issue, Yi et al. [39] sought to investigate a heterozygous *SHANK3* mutation and the wild-type alleles within the same genomic background in neuronal cells. For this purpose, they generated a conditional *SHANK3* mutation in human ESCs by deletion of exon 13 causing a frameshift in all major SHANK3 mRNAs, an experimental design matching the predicted outcome from nonsense de novo mutations of *SHANK3* in SCZ [68].

**Figure 4:**
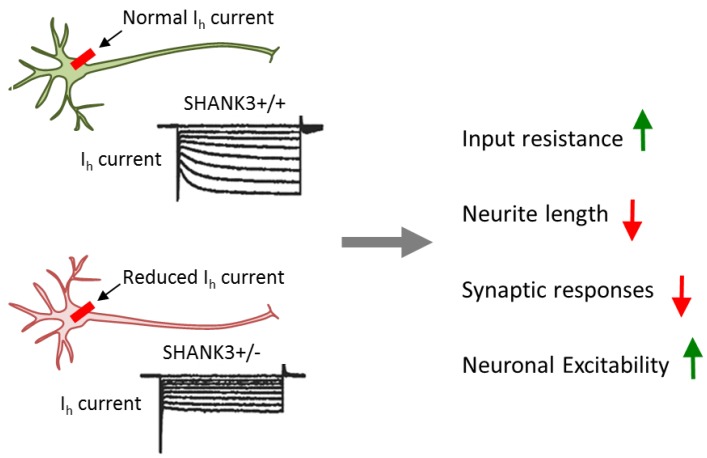
Conditional *SHANK3* deletion impairs *I*_h_ channel. Comparison of isogenic wild-type and *SHANK3-*deficient human ESC-derived excitatory iNs. Heterozygous and homozygous *SHANK3* mutations strongly reduce *I*_h_ channel function and gave rise to multifarious impairments including a decrease in dendritic arborization and synaptic responses and an increase in input resistance and neuronal excitability. Adapted from [39], attributable license 4707100181499.

Heterozygous conditionally mutant *SHANK3* ESCs were differentiated towards excitatory iNs in the presence of active or mutant Cre-recombinase to yield heterozygous mutant or wild-type neurons. *SHANK3* deletion in glutamatergic excitatory neurons caused impairments in dendritic arborization, intrinsic electrical properties, and synaptic transmission. These results resembled the ones from iPSC-derived neurons from patients with PMDS, thus strengthening the causal role of *SHANK3*. At the same time, they re-exposed the question of how impaired synaptic transmission links to increased input resistance (Figure 4).

Interestingly, blockage of HCN (hyperpolarization-activated cyclic nucleotide-gated) channels, but not of other ion-gated channels, strongly increased input resistance and resting potential in wild-type, but not in SHANK3 mutant neurons. HCN channels are encoded by four genes (*HCN1* to *HCN4*) and mediate hyperpolarization-activated *I*_h_ currents [70]. These currents facilitate membrane depolarization toward the action-potential threshold and reduce membrane resistance. Thus, *I*_h_ currents regulate neuronal excitability, network activity, and plasticity. Consistent with this role, chronic inhibition of HCN channels in differentiating wild-type neurons impaired neuronal morphology in a manner undistinguishable from *SHANK3* haploinsufficiency. Mechanistically, SHANK3 interacted directly via its ankyrin repeats with HCN channels and may enrich HCN channels at postsynaptic sites.

In sum, this study supports a causal role of *SHANK3* in multilayered synaptic dysfunction: haploinsufficiency impairs *I*_h_ channel function as a primary cause of intrinsic changes in neuronal properties. Electrophysiological and morphological alterations secondarily converge on synaptic dysfunction. This chain of events suggests that impairment in *I*_h_ current is a major pathogenic force of *SHANK3* mutations in predisposing to development-related psychiatric disorders like ASD and SCZ. Importantly, *I*_h_ channel dysfunction is an actionable target that can be pharmacologically manipulated in order to potentially ameliorate the neurobehavioral correlates of *SHANK3* mutations in patients.

Beyond synapses, SHANK3 is already expressed during early stages of neuralization in vitro. To explore SHANK3′s role during this period, Kathuria et al. [71] generated iPSCs from two patients with ASD carrying heterozygous microdeletions in *SHANK3* and from three healthy controls, as well as isogenic *SHANK3* knock-out ESCs. Pluripotent cells were differentiated into either cortical or olfactory placodal neurons and tracked during varying stages of development in vitro. Consistent with previous reports, mature cortical, but also olfactory placodal neurons from patients showed reduced SHANK3 and synaptic marker expression relative to controls. Notably, the reduction in SHANK3 expression in patient cells was significantly greater during neuralization than at synaptic stages. Concurrently, immature olfactory placodal neurons from patients exhibited smaller cell soma, and more and longer primary neurites, relative to control cells. By contrast, immature cortical neurons from patients revealed a reduction in neurite length when compared to controls. Importantly, lentiviral overexpression of SHANK3 rescued the morphogenetic phenotype in immature olfactory placodal neurons from patients and prevented later synaptic deficits. In complementary experiments, ESCs engineered to contain heterozygous or homozygous deletions of *SHANK3* phenocopied the deficits from patient-derived iPSCs, albeit at an earlier stage. 

In brief, this report [71] assigns to *SHANK3* a cell type-specific role in neural development. When viewed together with foregoing reports [38,39], this finding illustrates the need to investigate cause-effect relationships in iPSC-based modeling across different developmental periods and cell types in vitro to capture multilayered effects from causal variants in psychiatric disorders. 

## 4. ESC/iPSC-Based Modeling of Common GWAS Risk Variants and Genes

Our understanding of how common genetic risk variants causally contribute to psychiatric disorders is still little (Figure 1). Risk SNPs map typically to non-coding regions of the genome, such as intergenic and intronic regions, and capture all the genetic variation localized in a risk SNP-associated haplotype block [5]. Since risk-associated SNPs are on themselves not necessarily the causal regulatory element underpinning disease, the generation of gene-edited isogenic iPSC lines is an important step on the way to dissect the causal role of single SNPs on molecular and cellular phenotypes in living human neurons.

### 4.1. Credible SNP at miR-137 Regulates Dendritic Arborization 

Non-coding RNAs of ~70 nucleotides in size (pri-miRNAs) are cleaved by the nuclear DGCR8 (DiGeorge syndrome chromosomal region 8)/DROSHA protein complex into precursor RNAs (pre-miRNAs). These intermediates are further cleaved by the cytoplasmatic endoribonuclease DICER to yield single-stranded mature microRNAs (miRNAs) of ~22 nucleotides in size [72]. Finally, miRNAs are incorporated into the RISC complex (RNA induced silencing complex) and target through a 6- to 8-base pair complementary ‘seed region’ of one or more mRNAs to downregulate their expression. During brain development and beyond, miRNAs regulate cell lineage and fate decisions, neuronal differentiation, and maturation [73,74]. Consistent with this role, altered miRNA expression profiles were detected in postmortem brains from patients with SCZ, MD, and ASD [75]. Furthermore, supporting the primary role of miRNAs in psychiatric disorders, miRNA-137 (miR-137) maps to a risk locus in SCZ [76,77] and regulates other risk genes, like *CACNA1C* (calcium channel, voltage-dependent, l-type, alpha-1C subunit) or *TCF4* (transcription factor 4) [78,79]. 

To explore the role of *MIR137* for neural development in vitro, Siegert et al. [80] directly differentiated fibroblasts carrying either the major or minor allele (each encoding all four non-coding risk SNPs) in early neuron-like cells. Interestingly, miR-137 expression was significantly increased in the presence of the minor allele when compared to the major allele and more strongly downregulated presynaptic plasticity genes and neuronal activity-dependent synaptic vesicle release. Although which allele and non-coding SNPs actually contribute to disease risk was left unanswered by these experiments. 

**Figure 5:**
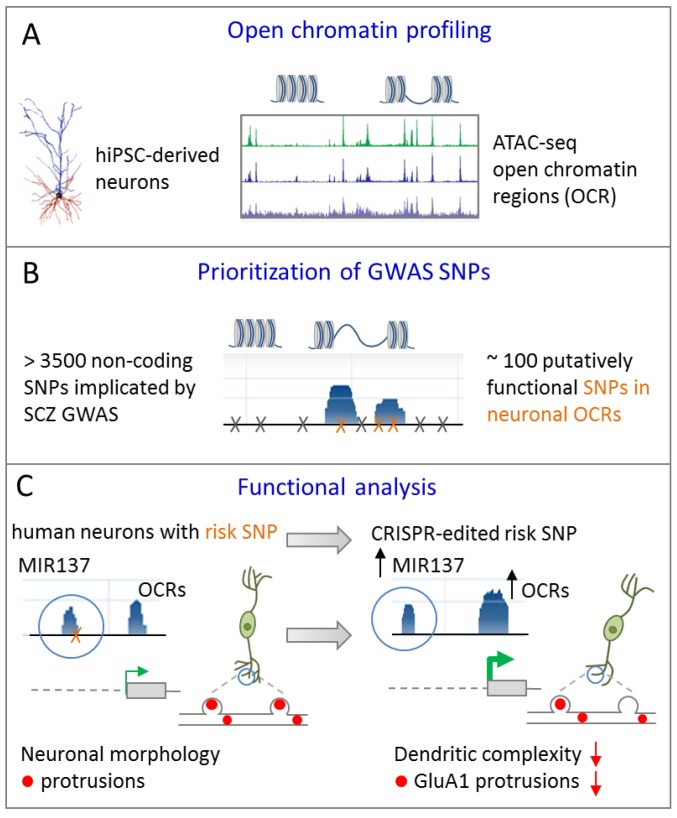
A common GWAS SNP at *MIR137* regulates dendritic arborization. (**A**) Assay for transposase-accessible chromatin sequencing (ATAC-seq) is used to define open chromatin regions (OCRs) during differentiation of human iPSCs towards excitatory forebrain neurons. (**B**) GWAS SNPs from SCZ are enriched in OCRs and are prioritized accordingly. (**C**) An isogenic iPSC line from a patient with SCZ differing solely at the predicted functional GWAS risk SNP in *MIR137* was generated by CRIPSR/Cas9 editing. When compared to the risk allele (left), conversion to a non-risk allele enhanced chromatin dynamics, leading to increased miR-137 expression in excitatory iNs (right). Presence of the risk allele associated with more mature dendritic arbors and neuronal protrusion of excitatory iNs relative to isogenic cells carrying the non-risk allele. Adapted from [40], attributable license 4695391223436.

In an independent approach, Forrest el al. [40] sought to prioritize non-coding GWAS risk variants using chromatin analysis in differentiating iPSCs. As evidenced by ATAC-sequencing (assay for transposase-accessible chromatin) open chromatin regions (OCRs) underwent dynamic changes during differentiation towards excitatory cortical neurons (Figure 5A).

Neuronal OCRs and TF binding sites were enriched for GWAS risk variants in SCZ and significantly narrowed down the number of putatively functional variants (Figure 5B). With respect to *MIR137*, one potentially functional common risk SNP (rs1198588) localized to a neuronal OCR (note that only a subset of known *MIR137* risk SNPs mapped to neuronal OCRs allowing for functional prioritization). To understand the functional relationship between this SNP and the overlaying ORC, and by implication of miR-137 expression, isogenic iPSC lines from a patient with SCZ differing solely at the predicted functional GWAS risk SNP were generated by CRISPR/Cas9 editing (Figure 5C). Modifying the risk SNP to a non-risk-allele affected chromatin dynamics at the *MIR137* locus and led to increased miR-137 expression in glutamatergic iNs. This increase in miR-137 expression associated with a reduced level of maturity of the dendritic arbor and of neuronal protrusions indicating altered developmental trajectories (Figure 5C). Contrariwise, presence of the GWAS risk SNPs led to reduced miR-137 expression and was associated with a more mature phenotype of glutamatergic neurons. 

Collectively, Forrest et al. [40] firstly defined a causal role for a common GWAS SNP in SCZ by using patient-specific isogenic iPSCs, in which the risk SNP was gene-edited to a non-risk allele. This allows comparing the effect of *MIR137* risk and non-risk alleles within the same genomic background on chromatin conformation, gene expression, and neuronal phenotypes in vitro. It must be kept in mind that this analysis does not exclude the possibility of additional *MIR137* SNPs with a causal role that may act in the same or opposite direction in cortical or other neuronal cells. In a broader context, it would also be interesting to study the effects from risk and non-risk alleles within the genomic background of healthy controls to assess potential interactions with other risk loci in SCZ. 

### 4.2. Joint Effects of Common GWAS Risk Variants on Gene Expression 

SCZ is a highly polygenic disorder involving many variants encoding for small effects [5]. The mechanisms through which these variants interact and ultimately lead to disease are largely unexplored. Therefore, Schrode et al. [41] most recently sought to perturb the expression of genes in close vicinity of several risk variants, singly or together, in isogenic iPSC-derived neuronal cell types. Common variants and genes from the current data set of SCZ-associated loci [81,82] were prioritized by integrating GWAS data and postmortem gene expression profiles to identify those variants that potentially affect the risk for SCZ via their effect on gene expression (genetic variants controlling gene expression levels in cis or in trans are called expression quantitative trait locus (cis- or trans-eQTL), respectively). eQTLs are thought to play an important role during early brain development in SCZ (for review [83]). Among 108 risk loci, 19 contained GWAS SNP and cis-eQTL within 1 Mb of a gene. Of these candidate loci, five were predicted to encode single protein-coding genes, namely *FURIN*, *TSNARE1*, *CNTN4*, *CLCN3,* and *SNAP91.* Briefly, *FURIN* encodes for a proprotein convertase that activates a variety of regulatory proteins in the constitutive exocytic and endocytic pathway; *TSNARE1* encodes for t-SNARE domain containing one with a possible role in intracellular protein transport and synaptic exocytosis; *CNTN4* encodes for contactin 4, an axon-associated cell adhesion molecule with roles in the formation, maintenance, and plasticity of functional neuronal networks; *CLCN3* encodes for chloride voltage-gated channel 3; and *SNAP91* encodes for synaptosomal-associated protein, 91-KD with a likely role in active synaptogenesis and presynaptic maturation. This gene set showed robust expression at different stages of neuronal differentiation in vivo and in excitatory iNs except *CNTN4*. 

To assess the role of the cis-eQTL in *FURIN’s* 3′ untranslated region, CRISPR/CAS9-mediated genetic editing (AA to GG) was applied to generate homozygous non-risk alleles in iPSC lines from healthy donors. Isogenic iPSCs were differentiated into excitatory iNs (via Ngn2), GABAergic iNs (via ASCL1/DLX2), and induced astrocytes (via NFIB, nuclear factor 1 B-type). Allelic conversion resulted in decreased *FURIN* expression in excitatory neurons but was without effect in GABAergic iNs or induced astrocytes, and unexpectedly, upregulated *FURIN* expression in isogenic NPCs. Consistent with FURIN’s role in the proteolytic activation of pro-nerve growth factor, isogenic NPCs carrying non-risk alleles exhibited increased neural migration relative to isogenic controls. Conversely, excitatory iNs carrying the non-risk alleles, leading to lower *FURIN* expression, showed reduced neurite length and neuronal activity.

Given that no single cis-eQTL for allelic conversion could be identified for *TSNARE1*, *CLCN3*, and *SNAP91*, Schrode et al. [41] opted for CRISPR/CAS9-mediated activation and inactivation (CRISPRa/i) of each credible gene in isogenic iPSC-derived cells. Since both *TSNARE1* and *SNAP91* are thought to regulate synaptic vesicle recycling, the researchers focused on CRISPRa/i manipulation of these two genes in excitatory iNs to dissect their synaptic impact through isogenic comparisons. When all synaptic phenotypes obtained from comprehensive immunohistochemistry and electrophysiology were summarized in an integrative heatmap, samples with SCZ-relevant upregulated perturbation in *SNAP9*1 and *TSNARE1* correlated only weakly to each other. By contrast, a remarkable congruence in the effects at the synapse level was detected between *SNAP91* CRISPRi and *TSNARE1* CRISPRa, which was also supported by gene enrichment patterns deduced from global gene expression profiling. These findings highlighted the complexity of possible consequences of SCZ-associated eQTLs and raised the possibility that subtle changes in target gene expression in either direction can converge on synaptic phenotypes (see Section 4.2 for further discussion).

In light of the highly polygenic nature of SCZ, Schrode et al. moved on to assess the effect of perturbation of the four credible genes singly or jointly in a disease-relevant direction (RNA interference mediated downregulation of *FURIN* together with CRISPRa upregulation of *TSNARE1*, *CLCN3,* and *SNAP91*) on the transcriptome of iPSC-derived NPCs from healthy donors. Interestingly, each single perturbation resulted in transcriptional effects opposite to disease-associated transcriptional changes as inferred from iPSC-derived neurons and postmortem tissues from patients with SCZ, while joint perturbation resulted in transcriptional effects concordant with disease associated transcriptional changes. Moreover, under joint perturbation, a subgroup of the upregulated (7%) and downregulated (11%) genes were more strongly regulated than expected by the additive effects of singly manipulated credible genes. Such synergistic gene regulation occurred particularly in genes relevant to pre- and postsynaptic functions, genes containing rare CNVs or non-synonymous de novo mutations in SCZ, and genes implicated in SCZ GWAS (see Section 4.2 for further discussion).

Taken together, Schrode et al. [41] integrated three complementary lines of experiments in the analysis of GWAS risk variants: (i) postmortem gene expression analysis to prioritize risk variants based on their effect on gene expression, (ii) genetic editing to investigate the causal role of a credible SNP in isogenic iPSC-derived cells, and (iii) CRISPRa/i-mediated regulation of credible genes to investigate polygeneticity in iPSC-derived cells. 

### 4.3. Cataloging Credible Risk SNPs and Genes 

Various approaches including OCR mapping in disease-relevant human neuronal cells [40] and cis-eQTL mapping in human postmortem brains [41] have been used to narrow down credible SNPs that may contribute to psychiatric disorders. Yet, on average these methods rarely accomplish single-SNP resolution, and even less, complete analysis of all credible SNPs. Alternatively, massively parallel reporter assays (MPRAs) present scalable, functional readouts to genetic variation: DNA sequence elements from each allele are inserted into a reporter plasmid and once transfected into cells, the promoter or enhancer activity of these elements is measured quantitatively [84,85,86,87,88]. As of yet, MPRA studies have covered only a tiny fraction of the genome. Therefore, van Arensbergen et al. [89] set up a modified version to survey the regulatory function of ~5.0 million SNPs, corresponding to about one half of known common SNPs, in two different non-neural cell types. Interestingly, ~30,000 SNPs altered the regulatory activity of enhancers or promoters with ~90% of the regulations taking place only in one of the two tested cell lines, which is consistent with previous eQTL-based studies [90]. This suggests that a significant proportion of all human regulatory SNPs may act in a cell-type specific manner. In this regard, MPRAs with a focus on iPSC-derived disease-relevant cell types are needed to capture functional variation among current risk variants from psychiatric disorders. There are also thousands of additional genetic variants that show strong association with psychiatric disorders just below the genome-wide significance cutoff. As it is very likely that many of these variants will cross this threshold with increasing cohort sizes, the need for functional analysis will increase in parallel. 

Critically though, Arensbergen et al. investigated the function of potential regulatory DNA elements in the context of a plasmid construct, a technical approach frequently used in MPRA-based studies. However, plasmids become only poorly chromatinized upon transfection into the host cells and thus appear less suited to inform on the regulation of higher order chromatin structure and long-range regulatory interactions [5]. This drawback becomes more demanding given that the nearest gene model, in which non-coding variants are assigned on a linear DNA strand to neighboring genes, is increasingly questioned by both experimental and computational evidence [91,92]. For example, *FTO* (encoding for α-ketoglutarate-dependent dioxygenase) is highly expressed in hypothalamic nuclei governing energy balance with its mRNA being regulated by feeding and fasting. Well-fittingly, two independent obesity-associated SNPs were found to map to *FTO*. Yet, these credible SNPs do not regulate *FTO*, but form long-range functional connections with *IRX3* in the brain and both *IRX3* and *IRX5* in adipocytes [93,94].

Consistent with this scenario, Hi-C mapping (an extension of 3C that is capable of identifying long-range interactions in an unbiased, genome-wide fashion) of the germinal zone, cortical, and subcortical plates revealed a widespread three-dimensional (3D) chromatin structure in gene regulation during human brain development [42,95]. By integrating chromatin contacts with non-coding SCZ GWAS risk variants, Won et al. identified multiple credible genes and pathways, including acetylcholine receptors, postsynaptic density, neuronal differentiation, and chromatin remodelers. As a case in point, credible SNPs located 20 kb upstream of *DRD2* (encoding the D2 subtype of the dopamine receptor targeted by antipsychotic drugs) interacted physically with the promoter of *DRD2* thus corroborating its role as risk gene in SCZ [42]. Other examples of credible genes that were neither the nearest gene, nor in LD with the credible SNPs, included *FOXG1*, *EMX1*, *TBR1*, *SATB2*, *CUX2*, and *FOXP1*, all of which share a role in corticogenesis and cortical lamination. To provide experimental evidence for Hi-C based functional predictions, a credible SCZ-associated SNP, located 760 kb away from its physical interaction partner *FOXG1*, was gene-edited in primary fetal progenitor cells. Following their differentiation into primary neurons, the credible SNP regulated *FOXG1* expression, but not the nearby *PRKD1* locus, indicating a regulatory role during human cortical development [42]. While this report provides persuasive evidence for credible SNP-directed long-range regulation of disease-relevant genes in SCZ, it lacks insight into the biological consequences associated with the risk alleles. From a technical perspective, we would also like to caution that both reports [42,95] relied on complex heterogeneous tissue in their Hi-C experiments that are likely to conceal tissue-specific effects.

In another recent iPSC-based study, Rajarajan et al. [43] showed by Hi-C mapping that neuron-specific chromatin contacts are significantly associated with risk for SCZ. Specifically, risk-associated chromosomal contacts were conserved between iPSC-derived NPCs from healthy human donors and human fetal cortical plate [42] for five of seven loci tested (*CHRNA2*, *EFNB1*, *MATR3*, *PDH*, and *SOX2*, but not *ASCL1* or *DRD2*). The regulatory function of these conserved risk-sequence bound conformations was collaborated by CRISPR-mediated epigenomic and genomic editing experiments on isogenic NPCs. These experiments showed that chromosomal contacts anchored in SCZ risk loci regulate target gene expression across hundreds of kilobases outside GWAS linkage disequilibrium blocks. These targets present additional risk genes in a 3D space that extends the biological activity of SCZ risk loci in disease.

**Figure 6:**
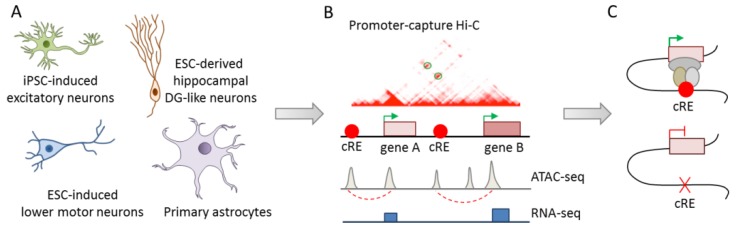
Charting the epigenomic landscape for regulatory variants. (**A**) Epigenomic mapping of promoter interacting regions (PIRs) was conducted in iPSC-derived excitatory iNs, ESC-derived lower motor iNs and dentate gyrus-like neurons, and primary fetal astrocytes. (**B**) PIRs identified by promoter capture Hi-C were enriched for active chromatin features and transcription as evidenced by ATAC-seq and RNA-seq, respectively. Additionally, PIRs were enriched in risk SNPs of major psychiatric disorders in a disease- and cell-type specific manner. (**C**) Monoallelic deletion of the PIR containing credible SNPs within the *DRD2* promoter led to downregulation of *DRD2* in excitatory cortical iNs. Adapted from [44], attributable license 4701990952085.

While Won and Rajarajan [42,43] exemplarily highlighted the causal role of credible SNPs in long-range gene regulation in iPSC-derived neuronal cells, integrated analysis of the large number of risk variants associated with psychiatric disorders is needed to dissect polygeneticity. Therefore, the development of scalable methods for charting the landscape of epigenomic regulation in well-characterized disease-relevant cell types could significantly advance the identification of causal variants. Towards this goal, Song el al. [44] conducted integrative analysis of chromatin interactions (promoter capture Hi-C), open chromatin regions (ATAC-seq), and transcriptomics (RNA-seq) in four functionally distinct human cell types: iPSC-derived excitatory iNs, ESC-derived lower motor iNs, ESC-derived dentate gyrus-like neurons, and primary fetal astrocytes (Figure 6A,B).

Overall, ~84% of the coding gene promoters (*N* = 17,065) participated in interactions in at least one cell type, ~40% among these interactions were cell type-specific, and ~80% took place within a distance of 160 kb. These numbers are likely to reflect transcriptional factories of coregulated genes, widespread colocalization of promoters, and the capacity of many promoters to doubly function as enhancers [44]. Functionally, promoter interacting regions (PIRs) were highly enriched for active chromatin features indicating a role in gene regulation, especially in cell fate commitment of neuronal cells. The identified chromatin interactions were next used to prioritize neuropsychiatric disorder- or trait-associated variants from the GWAS catalog [96]. Overall, PIRs were enriched in risk SNPs from major psychiatric disorders in a disease- and cell-type specific manner. SCZ SNPs were enriched at PIRs across all cell types: unipolar depression SNPs were enriched exclusively in excitatory and hippocampal dentate gyrus (DG)-like neurons, and BD SNPs showed also enrichment in lower motor neurons.

Exemplarily, the functional role of a PIR with credible SNPs at the *DRD2* promoter was assessed by CRISPR/CAS9-mediated monoallelic deletion in an iPSC line from a healthy donor. Following differentiation towards excitatory neurons, *DRD2* expression was downregulated in isogenic PIR-deleted cells relative to those carrying the wild-type alleles (Figure 6C).

In sum, this study takes us closer toward cataloging 3D epigenomic interactions in cell types relevant to psychiatric disorders and development. PIRs were enriched in risk variants from psychiatric disorders and preliminary evidence supports a causal role of PIRs in gene regulation. Critically though, the role of single risk SNPs within the PIRs, and by implication in chromatin interactions, remains unresolved. Relatedly, the integrative analysis of chromatin state and eQTL enrichment at PIRs relies on matched bulk tissues from the Roadmap Epigenomics Project and the Genotype-Tissue Expression (GTEx) project, respectively, thus preventing cell-type specific annotations for genomic and epigenomic features present at PIRs. Irrespective of these limitations, systematic prioritization of 3D regulation in disease-relevant cells provides an important handle to distinguish causative mechanisms from secondary phenotypes in highly complex brain tissues from patients with psychiatric disorders.

## 5. Outlook and Discussion

The identification of an increasing number of genetic variants urges the question of which of these variants causally contributes to psychiatric disorders, how they interact, and even more so of the functions they regulate. To meet this challenge, past studies focused on the role of rare high-risk CNVs in neural development and neuronal function in ESC-derived models. More recently, the advent of patient-specific iPSCs and (epi-) genetic editing catalyzed a shift from rare to common variants and their possible interactions in psychiatric disorders. This reorientation not only offers new possibilities, but also presents issues on interpretability. We will discuss the pros and cons of past and new research avenues, potential caveats, and upcoming developments in the field of ESC/iPSC-based modeling of causality in psychiatric disorders.

In the past, rare, highly penetrant CNVs presented a tangible entry point to uncover molecular and cellular mechanisms in psychiatric disorders. Guided by in vitro phenotypes from ESC/iPSC-based disease modeling and by potential correlates of neurobehavioral symptoms in patients, the list of candidate genes residing in the heterozygous deletions was empirically narrowed down. While this is a valid approach, current knowledge can bias the decision as to which genes are scrutinized in detail. Practically, the rescue of aberrant phenotypes through forced expression of candidate genes in neuronal cells carrying heterozygous risk alleles, and vice versa, induction of aberrant phenotypes through knockdown of candidate genes in neuronal cells carrying homozygous wild-type alleles, is used to inform on a causal role of individual genes in CNV driven pathology. While this experimental design often yields complementary results in vitro, matching to varying degrees those in vivo (e.g., postmortem brain analysis or animal studies), several caveats still come to mind. Typically, rescue and knockdown experiments of candidate genes rely on immediate effects outside the range of physiological expression levels that may override or phenocopy disease mechanisms without attesting to a causal role. This is a critical issue given the extraordinary plasticity of ESCs/iPSCs and their derivatives, and by implication, of neural development in vivo. In addition, the effect from manipulation of candidate genes on the expression of other genes contained in the deleted region, and importantly, on genome-wide expression profiles, needs to be carefully scrutinized. Ideally, heterozygous deletions ought to be reconstituted in their entirety in the absence and presence of a functional copy of the respective candidate gene. Presently, this claim poses a severe, if not insurmountable, technical challenge owing to the large size of most CNV driven deletions. An exception to this quandary is the CNV at 2p16.3 that impacts only one gene, *NRXN1*. This circumstance facilitated assigning a causal role in aberrant neural development and neuronal activity in vitro to heterozygous *NRXN1* deletions [31]. However, it remained unanswered as to what degree *NRXN1*-associated phenotypes depend on genomic context. Despite their high penetrance, CNVs, including 2p16.3, are found in healthy parents and siblings [29]. Comparative modeling of iPSCs derived from patients and unaffected relatives with CNVs could help gain insight into this topic. This is of particular interest for the early detection of those carriers at risk which may develop full-blown symptomatology. In this context we note that isogenic modeling of private CNVs in *PCDH15* and *RELN1* in iPSCs from healthy donors recapitulated some, but not all, features of patient-specific iPSCs [33]. Although this may be explained by gene-dosage effects due to heterozygous deletions in patient-specific iPSCs versus homozygous deletions introduced into iPSCs derived from healthy donors, patient-specific iPSCs seemed to compensate to a significant degree for allelic loss by upregulation of the remaining wild-type allele during neural differentiation in vitro. This intriguing finding raises the question as to what degree compensatory responses can accumulate during the course of development in vivo and whether they can be adequately captured by short term in vitro experiments. 

Unsurprisingly, many of above concerns apply as well to the study of common GWAS risk variants. In an elegant study, Forrest et al. [40] brought together prioritization and functional analysis of common variants during differentiating of iPSCs toward cortical fates. Interestingly, modifying the risk SNP to the non-risk allele caused reduced maturity of the dendritic arbor and of neuronal protrusions in vitro. This behavior of cortical gene-edited iNs seems at odds with reduced dendritic arborization in postmortem brains from patients with SCZ [97]. However, iPSC-derived neuronal cells match early developmental stages, whereas postmortem brain analysis reflects the aggregate of disease course, treatments, and life history at adult stages in patients of unknown *MIR137* status. In this regard, it is important to note that Forrest et al. investigated the causal role and the direction of the effects of the risk SNP in a disease-relevant polygenic background in vitro. Since we do not know whether, how, and to what degree single risk SNPs interact with other risk SNPs in the mediation of aberrant molecular and cellular phenotypes, care needs to be taken to assess their role in the appropriate genomic context. 

Moving beyond single SNPs, Schrode et al. [41] sought to define the interplay between different GWAS-credible SNPs in SCZ. Firstly, they showed that the *FURIN* cis-eQTL operates in a lineage- and cell-type specific manner in iPSC-based 2D culture systems. This finding is consistent with general principles of gene regulation [98] and strengthens the notion to investigate credible SNPs under various conditions to identify their regulatory role. Still, the regulatory role of the *FURIN* cis-eQTL was no longer significant when measured by quantitative PCR in glutamatergic and GABAergic spheroids. Since such 3D cell aggregates exhibit higher cellular heterogeneity than conventional 2D culture systems (see below), single cell analysis of spheroids appears more apt than bulk preparations to investigate subtle eQTL effects.

Testing epigenetic regulation of *SNAP9*1 and *TSNARE1,* two genes with a role in vesicle recycling, Schrode et al. [41] observed that subtle changes in cis-eQTL target-gene expression in either direction can converge on synapse phenotypes. A striking congruence was detected between synaptic effects caused by *SNAP91* downregulation (opposite to the direction in SCZ) and *TSNARE1* upregulation (in the direction of SCZ). By contrast, the synaptic effects caused by SCZ-relevant upregulation in *SNAP9*1 and *TSNARE1* correlated only weakly to each other. This outcome seemed to question unidirectional changes in cis-eQTL target-gene expression in postmortem brains from patients with SCZ. Some caveats need, however, to be considered. The fact that *SNAP91* downregulation and *TSNARE1* upregulation converged on similar synapse phenotypes requires cautious interpretation with respect to its relevance for SCZ. While any deviation from physiological SNAP91/TSNARE1 expression levels may disrupt the cellular logic of synaptogenesis and synaptic function, this does mean that they occur in living brains, where the nature of the genetic variation determines the direction of gene expression changes. In the absence of information on higher-level function (see below), it is not possible to decide whether congruent or incongruent effects from in vitro manipulations of risk genes are more critical to SCZ. Therefore, caution needs to be exercised to extrapolate from perturbation of candidate genes in vitro to disruption of cellular functions in vivo, or even higher brain functions in disease. While postmortem analysis remains important to the interpretation of in vitro results, it is equally important to realize that iPSC-based modeling reflects early stages of neural development in vitro that are more likely to inform on the predisposition to develop a psychiatric disorder than the disease state of the mature brain itself. [26].

Moreover, caution should be applied to the interpretation of the results from joint in vitro regulation of SCZ risk genes on gene expression profiles: disease-relevant perturbation of four credible genes in iPSC-derived neurons from healthy donors correlated positively to changes from iPSC-derived neurons and postmortem tissues from patients with SCZ, while single perturbation did not. One possible explanation of this puzzle is that the regulation of these risk genes is interdependent through mechanisms unknown at present. If this is true, then disruption of the transcriptional logic underpinning synapse function may lead to different outcomes between single or joint deregulation. Broadly, these observations illustrate the complex nature of SCZ genetic architecture, where single genetic perturbations/GWAS effects do not necessarily add up in a linear fashion. In fact, it is not surprising that complex regulatory cascades, potentially involving various types of feedback loops, especially during early stages of brain development, can operate downstream aggregated eQTL effects. Furthermore, it is important to note that these experiments were conducted in iPSCs derived from healthy donors that may promote outcomes different to those from patient-specific iPSCs, an important issue in light of the polygenetic nature of psychiatric disorders.

In an independent approach to single variants, recent endeavors on cataloguing SNPs en masse have gained momentum. These studies provide valuable estimates on the number of potentially functional SNPs, their tissue-specificity, and their role in long-range gene regulation. A remarkable, perhaps extreme, example of cell type specificity is the 20,000 or so neurons in the human hypothalamus that produce the neuropeptide orexin/hypocretin. Immune destruction of this tiny population of neurons leads to narcolepsy/cataplexy, a systematic human sleep disorder [99]. At this juncture, we know neither how many specialized categories of neuronal cells exist in the human brain (not to mention the daunting task to generate them from patient-specific iPSCs for disease modeling), nor how many risk-SNPs operate solely through this category of cells (not to mention specific developmental stages). In any case, further studies with a focus on psychiatry disorders, including genome-wide significant and subthreshold SNP associations, across different neuronal cell types, are looked for. In this context some principal limitations from aforecited Hi-C or promoter Hi-C studies should be mentioned: captured chromatin contacts can help assign putative target genes of a disease risk variant. However, chromatin contacts are also very dynamic and temporal (i.e., non-specific and stochastic). In fact, very few of the reported chromatin contacts are verified regarding their actual role in gene regulation. Moreover, chromatin contacts captured by Hi-C represent regional interactions, while a specific risk variant may have a very different functional effect from a chromatin region where it resides. For instance, a SNP may alter one or two TF binding site(s), but a DNA sequence region may mediate regulatory mechanisms well beyond the effect of a single TF at the SNP site. In light of these limitations, Fulco et al. [100] recently introduced a new technique (termed CRISPRi-FlowFISH) to investigate enhancer function and map candidate target genes in a single experiment. An important step forward for this method is that enhancer function, including the one from genetic variation, is measured on the basis of the expression of an endogenous candidate target gene, through fluorescence in situ hybridization (FISH). This technique could be applied in principle to any gene, and since it relies on endogenous genes rather than reporter constructs, candidate target genes are identified by definition. Since perturbation of gene expression is used as a proxy to enhancer function, this method also circumvents a critical assumption in chromatin-contact assays, in which proximity equals functional interaction. An important implication of this study [100] is that methods that infer enhancer function, and by implication genetic variation, on the basis of physical contact alone poorly predict actual regulatory connections and that chromatin contact, although important, portrays only part of the cellular regulome. Finally, we would like to point out that studying chromatin interactions in healthy cells allows the identification of regulatory interactions in the absence of disease-associated deregulations. However, biological pathway analysis in GWAS data has identified histone methylation processes among the processes that showed the strongest association across SCZ, MDD, and BD [9]. Hence, it is important to extend cataloging of risk SNPs to patient-derived cells as well as gain insights into how the 3D epigenome is altered in disease [101,102]. 

Notwithstanding recent progress on modeling causality in psychiatric disorders, it is important to keep in mind that at present, in vitro cultured cells can only approximate the cellular identity and complexity of the living brain. This shortcoming raises the question of what cellular phenotypes are needed to enhance identification of causal mechanisms and genes in psychiatric disorders. Previous studies on patient-specific disease modeling were directed towards gene expression profiling, imaging, electrophysiology, proteomic approaches, and functional readouts such as proliferation, migration, and maturation (for recent reviews see [103,104,105]. Qualitative and quantitative improvements on these readouts are likely to improve further reproducibility and statistical power from case/control studies. However, they may not necessarily accelerate the identification of causal mechanism and genes, or of causality at higher-levels of brain function. A widely-held misconception is that risk genes in psychiatric disorders encode for psychopathology—they do not. Risk genes are numerous with each variant conferring only subtle changes at the molecular and cellular level, mostly in a tissue-specific manner [12,16], which converge in the formation of micro- and macro-circuits during early brain development and beyond. Since behavior emerges from neuronal circuits processing information from the environment, aberrant synapse and circuit physiology is thought to lie at the core of psychopathology in psychiatric disorders.

Recent progress on compartmentalized microfluidic devices opened up the opportunity to model human brain circuitry in vitro with an unmatched level of control. Microfluidic chip devices physically segregate cell bodies of different neural subtypes while still allowing axonal growth between compartments [106]. At the same time, they are compatible with high-resolution video microscopy to investigate morphological and functional connectivity, to control axonal growth, and to monitor stages from early synaptic formation through late synaptic maturation. Hence, microfluidic chip platforms provide compelling systems to investigate causal mechanisms and genes in neural circuit dysfunction in psychiatric disorders.

As a proof of concept, Sarkar et al. [106] established a microfluidic chip platform to model the hippocampal dentate gyrus (hDG)-Cornu Ammonis region 3 (CA3) circuit of patients with SCZ. A two-compartment microfluidic device connected by narrow channels was used to seed pre-synaptic hDGs into one compartment and post-synaptic hCA3s into the opposing compartment. Synaptic connections formed within this microfluidic system as evidenced by a rabies virus tracing assay. Moreover, multi-electrode array (MEA) recordings and whole-cell patch clamp techniques showed that the SCZ hCA3 neurons were impaired in both spontaneous and evoked electrophysiological activity (see also [63]). 

Beyond 2D systems, advances in the field of brain organoids may also help to explore causal mechanisms and genes on cellular phenotypes relevant to psychiatric disorders. Brain organoids are 3D cell aggregates that recapitulate the cytoarchitecture of the developing brain more closely than conventional 2D culture. Brain organoids are produced in the presence of external patterning cues that drive differentiation into specific brain regions containing a broad repertoire of cell types well-matching their in vivo counterparts. However, brain organoids also exhibit considerable variability that can confound the investigation of case/control samples, and by inference, the identification of causal mechanisms [107]. In general, high organoid-to-organoid variability has raised concerns about the robustness of developmental trajectories outside the context of human embryogenesis. More recently, Velasco et al. [108] observed in an organoid model of dorsal forebrain that patterned organoids contain a wide repertoire of cell types that follow developmental trajectories from the human brain with similar variability, even though a particular challenge exists for models that need to produce brain regions from different regional lineages. For instance, the excitatory and inhibitory neurons of the cerebral cortex are derived from the germinal zones of the dorsal and ventral telencephalon, respectively. Application of dorsalizing patterning cues, however, will lead to the formation of an ‘all excitatory’ brain organoid with barely any inhibitory neurons. Alternatively, multiple cell lineages can be combined 3D in vitro to mimic the interactions between different cell lineages. Such aggregates, dubbed ‘brain spheroids’ allow improved insight into the formation of neural circuits and complex cell-to-cell interactions in vitro [109] that are particularly important to psychiatric disorders. 

In contrast to the significant advances in our understanding of the cellular diversity and morphological complexity of brain organoids, our insight into their electrical properties and neuronal connectivity remains limited at present. Extracellular recordings of brain organoids revealed spontaneous action potentials and coordinated bursting activity, supporting the development of functional neuronal networks [107]. Furthermore, nested oscillatory network activity has been measured within cortical organoids from six months onward in culture, indicating the potential to model the development of neural networks [110]. Beyond in vitro studies, transplanting of human brain organoids into mouse brains was recently performed to establish an in vivo model of human brain neuronal connectivity [111]. Human brain organoids underwent vascularization and integration with microglia, extended long-range axons, and exhibited synchronized neural activity and functional connectivity within the mouse brain (see also [63]). In a nutshell, human brain organoid in vitro and in vivo systems open up multiple opportunities to investigate causal mechanisms and genes on cellular phenotypes lying at the heart of psychiatric disorders. 

All in all, we are still far away way from a comprehensive census of causal variants in psychiatric disorders despite recent progress on iPSC-based disease modeling and (epi-) genetic editing. We have even less of a mechanistic understanding of how credible variants interact in the onset, manifestation, and course of psychiatric disorders. In the past years, credible variants were linked to deregulation of distinct molecular and cellular functions in neuronal cells in vitro. Yet, the human brain functions as a whole, not as the sum of its parts. Further steps are necessary to take these studies to higher level function analysis to gain insight into the behavioral, cognitive, and mood deficits in psychiatric patients. No single approach is likely to deliver final answers to the challenges outlaid here. However, the combination of different approaches from iPSC-based modeling, to animal studies, to deep patient phenotyping enable stepwise progress on our understanding of psychiatric disorders and light up new perspectives on future therapies.

## Figures and Tables

**Table 1:** Putative functional risk variants in psychiatric disorders supported by human embryonic stem cell/ induced pluripotent stem cell (hESC/iPSC)-based disease modeling.

Ref	Variation	Gene	System	Cell Type	Key Findings
[31]	CNV	*NRXN1*	Isogenic hESCS, conditional	Cortical glutamatergic	Impaired presynaptic neurotransmission, reduced spontaneous
	(2p16.3)		heterozygous mutations	iNS, cortical neurons	mEPSC frequency and EPSC amplitude
[32]	CNV	*CYFIP1*	Case/control-iPSCs	Neural rosette derived	Reduced adherens junctions and apical-basal polarity, gain- and
	(15q11.3)			NPCs	loss-of-function experiments in vitro and in mice support a causal
					role of CYFIP1
[33]	CNV	*PCDH15*	Case-control iPSCs, isogenic	EBs, glutamatergic and	Structural changes in iPSC-derived neurons from patients are
		*RELN1*	homozygous mutations in	GABAergic iNS	recapitulated by isogenic iPSC-derived neurons, heightened
			iPSCs from controls		AMPA and GABA receptor sensitivity in patients’ neurons
[34]	4-bp deletion	*DISC1*	Case-control iPSCs, isogenic	EB-derived mixed	Impaired presynaptic function in neurons derived from patient
			heterozygous mutation in	forebrain neurons	and gene-edited control iPSCs. Defect is rescued in neurons from
			control iPSCs, isogenic		gene-edited patient iPSCs. DEGs are enriched in genes important
			rescue in patient iPSCs		to synapse and neurodevelopment and psychiatric disorders
[35]	4-bp deletion	*DISC1*	Case/control-iPSCs	EB-derived mixed	Interaction between mutant DISC1 and ATF4 mediates aberrant
				forebrain neurons	gene expression that is rescued by forced ATF4 expression.
[36]	Exon 2 and	*DISC1*	Isogenic homozygous exon 2	EB-derived mixed	Mutations drive shift in dorsal fate owing to heightened Wnt-
	exon 8		or heterozygous exon 8	forebrain neurons	signaling. This promotes premature NPC differentiation and
			deletions		impaired cortical layer formation. Rescue by Wnt-antagonists
[37]	[34,36]	*DISC1*	See [34,36]	Cortical glutamatergic	Both mutations converge on few DEGs including UNCD5.
				iNS	Reduced neurite outgrowth is rescued by UNCD5 activation and
					phenocopied by UNCD5 knockdown in wild-type neurons
[38]	deletion	*SHANK3*	Case/control-iPSC	Mixed forebrain	Increased input resistance and impaired excitatory transmission
				neurons	associate with less excitatory synapses and glutamate receptors.
					SHANK3 complementation rescues only impaired transmission.
[39]	deletion	*SHANK3*	Isogenic hESCs, conditional	Cortical glutamatergic	Impaired synaptic transmission, dendritic arborization, and intrin-
			heterozygous mutations	iNS	sic electrical properties owing to perturbed interaction
					between SHANK3 and HCN channels in the postsynaptic density
[40]	SNP	*MIR137*	Isogenic iPSC from patient	Cortical glutamatergic	Credible SNP reduces miR-137 expression and leads to a more
			with SCZ	iNS	mature neuronal phenotype in vitro compared to the gene-edited
					non-risk allele
[41]	SNP	*FURIN,*	Isogenic iPSCs from healthy	Cortical glutamatergic	Credible SNP in *FURIN* reduces neurite length and neuronal
		*TSNARE1*	donors	iNs (NPCs and mature	activity in excitatory iNs. CRISPRa/i regulation of multiple risk
		*CLCN3*		neurons), GABAergic	genes converges on synaptic abnormalities, shows synergistic
		*SNAP91*		iNs, induced astrocytes	effects, and recapitulates changes from SCZ postmortem brains
[42]	SNP	*FOXG1*	Primary fetal progenitors	Primary fetal neurons	Credible SNP 760 kb away from *FOXG1* regulates FOXG1
					expression via chromatin contact
[43]	SNP		Isogenic iPSC from healthy	Cortical NPCs	Credible SNPs in GWAS loci regulate gene expression of *ASCL1*,
			donors		*EFNB1*, and *MATR3* via long range chromatin interaction
[44]	SNP		Isogenic iPSC from healthy	Excitatory and lower	PIRs are enriched for genes with a role in cell fate commitment
			donors	motor iNS, dendate	and for risk SNPs from major psychiatric disorders in a disease-
				gyrus like neurons,	and cell type-specific manner. Monoallelic deletion of the PIR at
				primary fetal astrocytes	*DRD2* causes downregulation in excitatory iNs
[45]	CNV	*NRXN1*	Case-control iPSCs	Glutamatergic (*NGN2*)	Heterozygous *NRXN2* deletions cause quantitative and qualitative
	(2p16.3)			and GABAergic	perturbations in isoform expression that associate with impaired
				*(ASCL2/DLX2)* iNS	neuronal and synaptic function. De novo isoforms arising from
					*NRXN1* deletions act potentially in a dominant negative mode.
					

Abbreviations: AMPA, α-amino-3-hydroxy-5-methyl-4-isoxazolepropionic acid; CRISPRa/i, CRISPR (clustered regulatory interspaced short palindromic repeats) mediated gene activation or inhibition; EB, embryoid body; DEG, differentially expressed gene; GABA, γ-aminobutyric acid; HCN, hyperpolarization-activated cyclic nucleotide-gated channel; hESC, human embryonic stem cell; iN, induced neuron; NPC, neural progenitor cell; mEPSC, miniature excitatory postsynaptic current; PIR, promoter interacting region; SCZ, schizophrenia; Wnt, wingless; GWAS, genome-wide association studies; SNP, single nucleotide polymorphisms.

## Abbreviations

3D	Three dimensional
ADHD	Attention deficit hyperactivity disorder
ATAC-seq	Assay for transposase-accessible chromatin
ASD	Autism spectrum disorder
BD	Bipolar disorder
CASK	Calcium/calmodulin-dependent serine protein kinase
CLCN3	Chloride voltage-gated channel 3
CNV	Copy number variation
CNS	Central nervous system
CNTN4	Contactin 4
COS	Childhood onset of schizophrenia
CRISPR/CAS9	Clustered regulatory interspaced short palindromic repeats/crispr-associated protein 9
CRISPRa/i	CRISPR mediated gene activation or inhibition
CYFIP1	Cytoplasmatic FMR1-interacting protein
DEG	Differentially expressed gene
DRD2	Dopamine receptor D2 subtype
EB	Embryoid body
EPSC	Excitatory postsynaptic current
eQTL	Expression quantitative trait locus
ESC	Embryonic stem cell
FURIN	Paired basic amino acid cleaving enzyme
FOXG1	Forkhead box G1
FTO	a-Ketoglutarate-dependent dioxygenase
GABA	γ-aminobutyric
GWAS	Genome-wide association study
iPSC	Induced pluripotent stem cell
iN	Induced neuron
NPC	Neural progenitor cells
NRXN1	Neurexin-1
MDD	Major depressive disorder
MRPA	Massively parallel reporter assays
mEPSC	Miniature excitatory postsynaptic current
OCR	Open chromatin region
PCDH	Protocadherin
PIR	Promoter interacting region
PMDS	Phelan-McDermid syndrome
PSD	Postsynaptic density
RELN	Reelin
RGC	Radial glia cell
RNA-seq	RNA sequencing
SHANK	SH3 and ankyrin repeat domain
SNAP91	Synaptosomal-associated protein 91-Kd
SNP	Single nucleotide polymorphism
SCZ	Schizophrenia
TF	Transcription factor
TSNARE1	t-SNARE domain containing 1
